# Effect of percutaneous renal denervation on blood pressure level and sympathetic activity in a patient with polycystic kidney disease

**DOI:** 10.1007/s00392-013-0647-1

**Published:** 2013-12-10

**Authors:** Aleksander Prejbisz, Jacek Kądziela, Jacek Lewandowski, Elżbieta Florczak, Ewa Żylińska, Mariusz Kłopotowski, Adam Witkowski, Andrzej Januszewicz

**Affiliations:** 1 Department of Hypertension, Institute of Cardiology, Alpejska 42, 04-628 Warsaw, Poland; 2 Department of Interventional Cardiology and Angiology, Institute of Cardiology, Warsaw, Poland; 3 Department of Internal Medicine, Hypertension and Angiology, Medical University of Warsaw, Warsaw, Poland; 4 Clinical Department of Noninvasive Cardiology and Arterial Hypertension, Central Clinical Hospital Ministry of Interior in Warsaw, Warsaw, Poland

Sirs:

The underlying mechanisms for the rise in blood pressure in individuals with autosomal dominant polycystic kidney disease (ADPKD) are complex, and experimental and clinical data support the concept that sympathetic hyperactivity may contribute to the pathogenesis of hypertension in ADPKD [1, 2].

More recently, a catheter-based percutaneous aimed at ablating both efferent and afferent renal nerve fibres has been introduced into clinical medicine and has been demonstrated to safely and effectively reduce blood pressure in patients with treatment resistant hypertension and normal renal function [3–6]. However, to our knowledge there is only one report of Shetty et al. [7] who observed after renal denervation resolution of chronic pain related to renal cysts and delayed fall in blood pressure in patient with ADPKD and resistant to treatment hypertension. No data on reduction of sympathetic activity has been shown.

A 26-year-old male with polycystic kidney disease was referred to the Department of Hypertension by the end of 2012 for the consideration of percutaneous renal denervation due to resistant hypertension. At the age of 20, the patient was first diagnosed with hypertension and also with ADPKD. A family history of polycystic kidney disease was present in the mother and in addition to in a sister.

At the time of referral, the patient had an office blood pressure of 162/77 mmHg and an average day-time ambulatory blood pressure of 138/73 mmHg despite four antihypertensive medications comprising valsartan 160 mg od, amlodipine 10 mg od, nebivolol 2.5 mg od, and hydrochlorothiazide 25 mg od. No history of cardiovascular disease or diabetes was reported, the patient has never smoked. The patient has normal renal function at baseline with a serum creatinine of 74 μmol/L and estimated glomerular filtration rate by MDRD formula of >60 mL/min/1.73 m^2^. The patient was not troubled by chronic flank pain.

On Doppler duplex examination renal arteries were normal, renal resistive index values were 0.61 and 0.66 in the right and left kidney, respectively. Abdominal ultrasound showed enlarged size of right (145 mm) and left (180 mm) kidney with multiple simple renal cortical cysts with a maximum diameter of 3 cm. Hepatic cysts were not present. Abdominal MRI showed bilateral simple appearing renal cysts in the upper and lower poles.

Microneurography was performed before and 3 months after the renal denervation. Muscle sympathetic-nerve activity (MSNA) signals were recorded by an electrode placed into the peroneal nerve at the popliteal fossa, posterior to the fibular head and the reference electrode was placed subcutaneously 2–3 cm from the recording electrode.

Due to his uncontrollable blood pressure percutaneous renal sympathetic denervation using the Symplicity Catheter^®^ system was performed as described previously [5], with five ablations in left and six in right renal artery (Fig. 1). The patient was discharged the following day on his pre-ablation antihypertensive regimen. The only complication of the procedure was transient stenosis of both arteries resulting from artery spasm and edema.

**Fig. 1:**
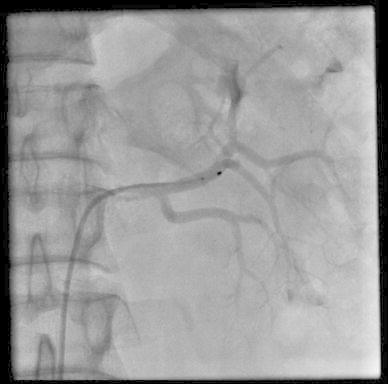
Symplicity Catheter^®^ in the left renal artery. It was possible to perform five ablations in the left renal artery

The patient was reviewed at 1 and 3 months post renal sympathetic denervation. At 1 month follow up he had improvement in his systolic blood pressure with an office reading of 129/60 mmHg and at 3 month follow up his blood pressure had dropped to 120/74 mmHg. A 24-h ambulatory blood pressure monitoring at 1 and 3 months confirmed blood pressure control with a mean day-time blood pressure of 131/68  and 128/65 mmHg, respectively. His renal function remained unchanged at 1 and 3 months. Microneurography at 3 months showed a reduction in MSNA (Fig. 2), as assessed in the peroneal nerve.

**Fig. 2:**
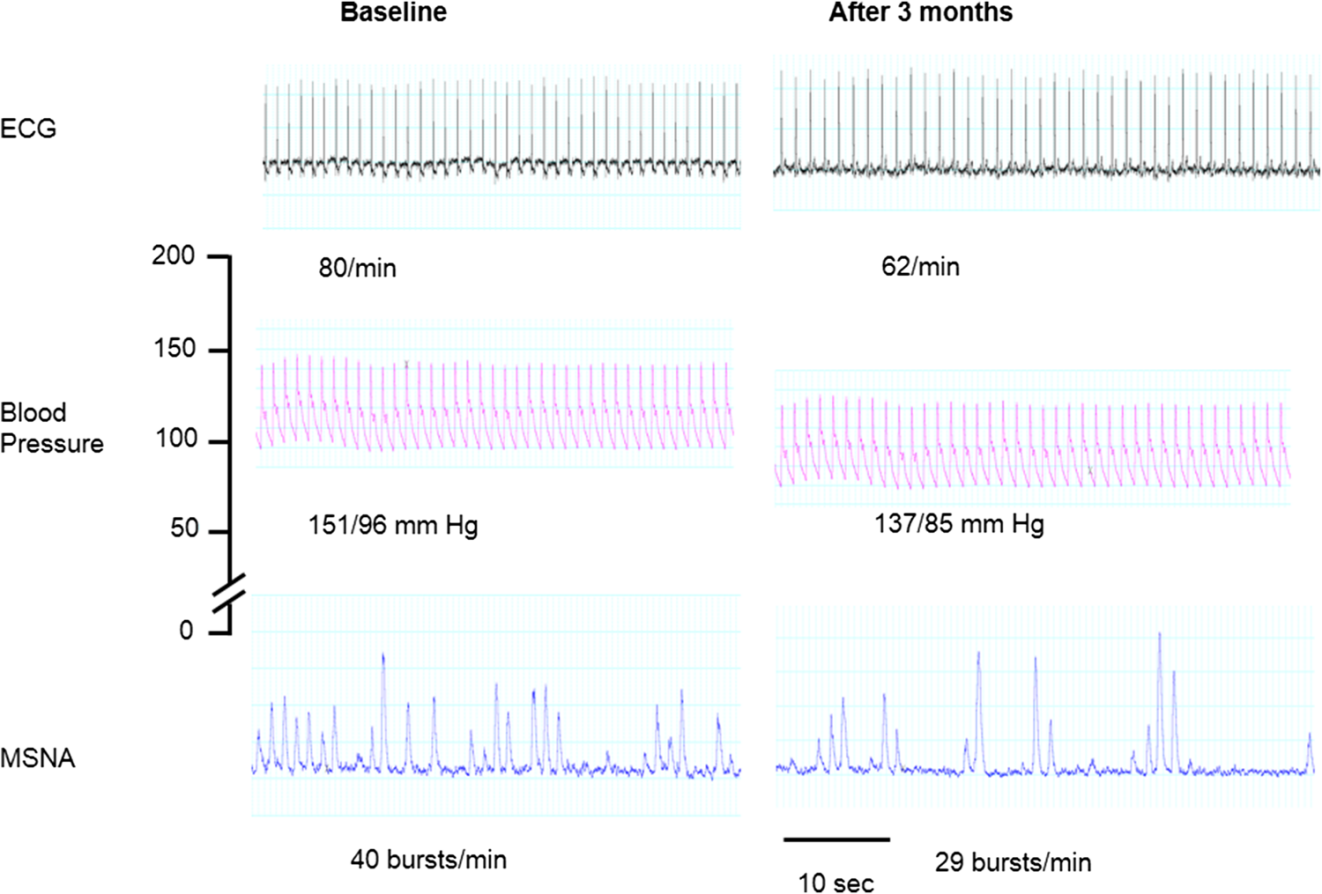
Reduction in blood pressure and muscle sympathetic-nerve activity (MSNA), as assessed in the peroneal nerve on microneurography 3 months after bilateral renal-nerves ablation

Our case report confirms the safety and effectiveness of a catheter-based renal denervation approach for the treatment of resistant hypertension in patients with ADPKD. While a single case has its obvious limitations, the fall in blood pressure after the procedure was accompanied by the decrease in the sympathetic activity being likely mediated via ablations of different fibres responsible. However, it should be mentioned that MSNA may not give information specifically about renal sympathetic activity [8].

In our case, larger reductions in office than in ambulatory blood pressure were noticed. However, these changes were of comparable magnitude as reported in the prospective studies [5, 9, 10]. In these groups, also the discrepancy between reduction in office and ambulatory blood pressure was also noted, but is should be stressed that observed reductions provide an equivalent reduction in cardiovascular events [5, 8, 11].

Confirmation of substantial BP lowering via catheter-based renal afferent denervation in patients with ADPKD in future studies may provide a valuable and safe alternative for the management of this difficult clinical condition.

## References

[1] Klein IH, Ligtenberg G, Oey PL, Koomans HA, Blankestijn PJ (2001). Sympathetic activity is increased in polycystic kidney disease and is associated with hypertension. J Am Soc Nephrol.

[2] Gattone VH, Siqueira TM, Powell CR, Trambaugh CM, Lingeman JE, Shalhav AL (2008). Contribution of renal innervation to hypertension in rat autosomal dominant polycystic kidney disease. Exp Biol Med (Maywood).

[3] Patel HC, Dhillon PS, Mahfoud F, Lindsay AC, Hayward C, Ernst S, Lyon AR, Rosen SD, di Mario C (2013) The biophysics of renal sympathetic denervation using radiofrequency energy. Clin Res Cardiol. [(Epub ahead of print) PubMed PMID: 24077678] 10.1007/s00392-013-0618-6 24077678

[4] Rippy MK, Zarins D, Barman NC, Wu A, Duncan KL, Zarins CK (2011). Catheter-based renal sympathetic denervation: chronic preclinical evidence for renal artery safety. Clin Res Cardiol.

[5] Esler MD, Krum H, Sobotka PA, Schlaich MP, Schmieder RE, Böhm M (2010). Renal sympathetic denervation in patients with treatment-resistant hypertension (The Symplicity HTN-2 trial): a randomised controlled trial. Lancet.

[6] Sobotka PA, Mahfoud F, Schlaich MP, Hoppe UC, Böhm M, Krum H (2011). Sympatho-renal axis in chronic disease. Clin Res Cardiol.

[7] Shetty SV, Roberts TJ, Schlaich MP (2013). Percutaneous transluminal renal denervation: a potential treatment option for polycystic kidney disease-related pain?. Int J Cardiol.

[8] Neumann J, Ligtenberg G, Klein II, Koomans HA, Blankestijn PJ (2004). Sympathetic hyperactivity in chronic kidney disease: pathogenesis, clinical relevance, and treatment. Kidney Int.

[9] Mahfoud F, Ukena C, Schmieder RE, Cremers B, Rump LC, Vonend O, Weil J, Schmidt M, Hoppe UC, Zeller T, Bauer A, Ott C, Blessing E, Sobotka PA, Krum H, Schlaich M, Esler M, Böhm M (2013). Ambulatory blood pressure changes after renal sympathetic denervation in patients with resistant hypertension. Circulation.

[10] Vogel B, Kirchberger M, Zeier M, Stoll F, Meder B, Saure D, Andrassy M, Mueller OJ, Hardt S, Schwenger V, Strothmeyer A, Katus HA, Blessing E (2013) Renal sympathetic denervation therapy in the real world: results from the Heidelberg registry. Clin Res Cardiol. [(Epub ahead of print) PubMed PMID: 24126436] 10.1007/s00392-013-0627-5 24126436

[11] Schmieder RE, Ruilope LM, Ott C, Mahfoud F, Böhm M (2013) Interpreting treatment-induced blood pressure reductions measured by ambulatory blood pressure monitoring. J Hum Hypertens. [(Epub ahead of print) PubMed PMID: 23636009] 10.1038/jhh.2013.39 23636009

